# Correlation Between COPD Assessment Test (CAT) Scores and the BODE Index in Stable Chronic Obstructive Pulmonary Disease (COPD): A Prospective Observational Study

**DOI:** 10.7759/cureus.107855

**Published:** 2026-04-28

**Authors:** Atharva Barve, Gaurav Sahu, Siddhi Rane, Ankita Biswas, Farhan F Bodha, Prisha Pranav Mehta, Pradip Potdar

**Affiliations:** 1 Department of Respiratory Medicine, Mahatma Gandhi Mission (MGM) Medical College and Hospital, MGM Institute of Health Sciences, Navi Mumbai, IND; 2 Department of General Medicine, Mahatma Gandhi Mission (MGM) Medical College and Hospital, MGM Institute of Health Sciences, Navi Mumbai, IND

**Keywords:** bode index, cat score, clinical correlation, copd, disease burden, fev₁, resource-limited settings, spirometry

## Abstract

Background

Chronic obstructive pulmonary disease (COPD) remains a leading cause of global morbidity and mortality, yet its clinical assessment continues to rely heavily on spirometry, which inadequately reflects patient-centred disease burden. The need for rapid, scalable, and clinically meaningful assessment tools is particularly critical in resource-constrained settings. This study explores whether the COPD Assessment Test (CAT), a simple symptom-based instrument, correlates strongly with multidimensional prognostic indices such as the Body mass index, airflow Obstruction, Dyspnea, and Exercise capacity (BODE) index.

Methods

A prospective observational study was conducted on 60 stable COPD patients. Clinical, demographic, and exposure data, including smoking and biomass exposure, were systematically recorded. All patients underwent spirometry and assessment using the CAT score and the BODE index. Correlation analysis using Pearson’s coefficient was performed to evaluate relationships between the CAT score, BODE index, and forced expiratory volume in one second (FEV_1_)​​​​​​.

Results

The study cohort demonstrated a high burden of disease with predominant moderate-to-severe airflow limitation. Risk factor analysis revealed substantial exposure to both smoking and biomass fuels, reflecting real-world environmental contributions to COPD. Symptomatically, breathlessness and productive cough were highly prevalent, indicating advanced disease at presentation. A strong positive correlation was observed between CAT score and BODE index (r = 0.808, 95% CI: 0.69-0.89, R^2 ^= 0.653, p < 0.001), suggesting close alignment between patient-reported outcomes and multidimensional disease severity. Additionally, the CAT score showed a strong inverse correlation with FEV_1_ (r = -0.729, 95% CI: -0.90 to -0.71, R^2 ^= 0.687, p < 0.001), comparable to the relationship between the BODE index and FEV_1_ (r = -0.73, p < 0.001). These findings demonstrate that the CAT score captures both symptomatic and physiological dimensions of COPD.

Conclusion

This study demonstrates that the CAT score, a rapid and easily deployable tool, strongly correlates with the BODE index and spirometric severity. These findings suggest that CAT may complement comprehensive COPD assessment as a complementary screening tool, particularly in resource-limited healthcare settings. However, CAT should not be considered interchangeable with the BODE index, which captures additional dimensions such as body mass index and formal exercise capacity. Further validation in diverse populations is needed before routine clinical implementation.

## Introduction

Chronic obstructive pulmonary disease (COPD) is a common, preventable, and treatable respiratory disease characterised by persistent respiratory symptoms and airflow limitation resulting from airway and/or alveolar abnormalities, typically caused by prolonged exposure to harmful particles or gases [1]. Due to its high prevalence, morbidity, and mortality, COPD represents a major global public health burden with significant socioeconomic consequences [2].

A systematic review and meta-analysis by Adeloye et al. estimated the global prevalence of COPD to be approximately 11.7% based on spirometric criteria [3]. According to the World Health Organisation, more than three million deaths annually are attributed to COPD, with the majority occurring in low- and middle-income countries [4]. COPD is projected to become the third leading cause of death worldwide in the coming years [4]. The Burden of Obstructive Lung Disease (BOLD) study reported a prevalence of 8.5% in women and 11.8% in men [5].

COPD is characterised by chronic airflow limitation resulting from an abnormal inflammatory response in the airways, lung parenchyma, and pulmonary vasculature [6]. The primary risk factors include tobacco smoking, occupational exposure to dust and chemicals, and environmental pollutants, including biomass fuel exposure [7-10]. Pathologically, COPD encompasses chronic bronchitis and emphysema. Chronic bronchitis is defined by chronic productive cough for at least three months over two consecutive years, whereas emphysema is characterised by destruction of alveolar walls leading to permanent enlargement of distal airspaces [11-13].

The Global Initiative for Chronic Obstructive Lung Disease (GOLD) 2025 report further emphasises the heterogeneity of COPD and expands disease classification to include entities such as pre-COPD and preserved ratio impaired spirometry (PRISM), highlighting early disease identification and the need for proactive management strategies [1]. Pre-COPD refers to individuals with respiratory symptoms and/or structural abnormalities without airflow obstruction, while PRISM is defined as reduced forced expiratory volume in one second (FEV_1_) with a preserved FEV_1_/forced vital capacity (FVC) ratio [1].

Despite its significant burden, COPD remains underdiagnosed worldwide. Lamprecht et al. demonstrated that up to 81.4% of COPD cases remain undiagnosed when assessed using spirometry [14]. In tertiary centres serving biomass-exposed populations in India, formal Body mass index, airflow Obstruction, Dyspnea, and Exercise capacity (BODE) index calculation is often impractical due to: (1) limited access to standardised six-minute walk test facilities; (2) time constraints in outpatient clinics; (3) high occupational COPD burden (58% of our cohort had biomass exposure), requiring rapid risk stratification. A simpler, scalable symptom assessment tool could improve early detection and monitoring of disease progression in these settings [15,16].

Although spirometry is the gold standard for diagnosis, it correlates poorly with symptom severity [17-19]. To overcome these limitations, multidimensional indices such as the BODE index, comprising body mass index, airflow obstruction, dyspnea, and exercise capacity, have been developed and shown to be superior predictors of mortality compared to FEV_1_ alone [20]. However, its use is limited in routine practice due to the requirement for exercise testing.

The COPD Assessment Test (CAT), a simple eight-item symptom-based questionnaire, has shown strong correlations with spirometry (r = 0.60-0.80) in Western populations [21]. However, the relationship between the CAT score and the BODE index, the multidimensional prognostic index that remains unexplored, particularly in resource-limited settings with high occupational COPD burden, is addressed in this study.

Study hypothesis

We hypothesised that the CAT score would show a strong positive correlation with the BODE index (r > 0.70) and a strong negative correlation with FEV_1_ (r < -0.70), supporting its utility as a complementary assessment tool alongside traditional multidimensional indices in resource-limited settings.

The study aims to evaluate and compare the CAT score with the BODE index in patients with stable COPD. It seeks to measure both CAT scores and BODE index, analyse their correlation, and assess how each relates to spirometric parameters, particularly FEV_1_.

## Materials and methods

A prospective observational study was conducted over a period of 18 months in the Department of Respiratory Medicine at MGM Medical College and Hospital, Navi Mumbai. The study included 60 patients diagnosed with COPD, recruited from both outpatient and inpatient settings.

Patients were enrolled based on diagnostic criteria outlined in the GOLD 2025 guidelines, which include clinical history, physical examination, and spirometric confirmation of airflow limitation (post-bronchodilator FEV_1_/FVC ratio < 0.70). Only clinically stable patients were included in the study.

Sample size

Sample size calculation: For correlation studies, the sample size was calculated using the formula:

$n=\frac{(Z_{\alpha/2}+Z_{\beta})^2(1-r^2)}{\ln^2\!\left(\frac{1+r}{1-r}\right)}$, where Z_α/2_ = 1.96 (two-tailed α = 0.05), Z_β_ = 0.84 (β = 0.20, power = 80%), and r = expected correlation coefficient.

We assumed a moderate-to-strong correlation (r ≥ 0.70) between CAT and the BODE index based on prior findings. Using r = 0.70, the minimum required sample size was n = 54. To account for potential dropouts (5-10%), we recruited n = 60 participants, providing 80% statistical power to detect correlations ≥ 0.70 as statistically significant at p < 0.05.

Patients were included in the study if they were aged between 18 and 70 years, had a diagnosis of stable COPD without acute exacerbation at the time of evaluation, and provided written informed consent. Patients were excluded if they presented with an acute exacerbation of COPD; had significant comorbid conditions such as severe cardiac, renal, or hepatic disease or severe anaemia; or had other chronic respiratory diseases, including bronchial asthma, bronchiectasis, or interstitial lung disease. Patients who were unwilling to participate in the study were also excluded.

All patients with stable COPD attending the Department of Respiratory Medicine outpatient clinic or admitted to the inpatient ward during the 18-month study period (March 2024-August 2025) who met inclusion/exclusion criteria were prospectively identified via clinic schedules and admission records. Consecutive sampling was employed: e.g., Eligible patients were enrolled sequentially during each clinic day until the target sample of n = 60 was achieved. Screening log indicated X patients assessed, Y enrolled, Z ineligible (reasons: acute exacerbation n = A, comorbidities n = B, declined consent n = C). Written informed consent was obtained before any study procedures.

A standardised predesigned proforma was used to collect detailed demographic and clinical information, including age, gender, occupation, smoking history (type, duration, and pack-years), biomass exposure, and environmental or occupational risk factors. Clinical symptoms such as cough, expectoration, dyspnea, chest tightness, and fever were documented.

All patients underwent a detailed general and systemic examination. Baseline investigations, including chest radiography and pulmonary function tests, were performed. All spirometry was performed using the Jaeger Masterscreen PFT Pro (CareFusion, San Diego, USA) and conducted according to American Thoracic Society/European Respiratory Society (ATS/ERS) guidelines. Procedure included: (1) pre-bronchodilator and post-bronchodilator testing (albuterol 400 µg via metered-dose inhaler with spacer); (2) at least three acceptable manoeuvres per patient with reproducibility criteria (FEV₁ and FVC variation ≤ 150 mL); (3) quality control criteria including proper patient effort and smooth flow curves; and (4) daily equipment calibration per manufacturer specifications. Parameters recorded: forced expiratory volume in one second (FEV_1_), FVC, FEV_1_/FVC ratio, total lung capacity (TLC), and diffusing capacity of the lungs for carbon monoxide (DLCO), expressed as % of predicted values.

Six-minute walk distance (6MWD) was conducted on a 45-meter straight hospital corridor following ATS guidelines. Standardised verbal instructions were provided: "Walk as far as possible in 6 minutes; you may slow down or stop if needed." Continuous encouragement was provided every minute. Resting and post-test heart rate, oxygen saturation (SpO_2_), and blood pressure were recorded. Supplemental oxygen was administered if SpO_2_ fell below 88% during the test. The total distance walked in meters was recorded.

Assessment tools

The following tools were used for evaluation:

CAT Score

An eight-item validated questionnaire assessing cough, sputum, chest tightness, dyspnea, activity limitation, confidence, sleep, and energy. The questionnaire was administered by trained respiratory research assistants in the local language with standardised instructions. Patients completed the questionnaire independently without clinician input. Scoring: each item scored 0-5 points; total CAT score range 0-40. Interpretation: CAT ≥ 10 indicates symptomatic disease; ≥ 30 indicates high symptom burden. The CAT is a trademark of GlaxoSmithKline. Permission for use in academic research is not required.

BODE Index

A multidimensional scoring system integrating: B = body mass index (BMI); O = airflow obstruction (FEV_1_ % predicted); D = dyspnea (modified Medical Research Council (mMRC) scale); E = exercise capacity (6MWD). BODE index was calculated using the validated formula by Celli et al. [20], with scores ranging from 0 to 10. Higher BODE scores indicate greater disease severity and mortality risk.

Assessment independence and blinding

Assessments were conducted independently to minimise potential bias. Spirometry assessors were not aware of CAT scores at the time of spirometry testing. Similarly, CAT administrators did not have access to spirometry results when administering the questionnaire. This separation of assessments reduces the risk of one assessment influencing the other, though complete double-blinding was not feasible given the open-label nature of this observational study.

Statistical analysis

Data were analysed using IBM SPSS Statistics for Windows, Version 27 (Released 2019; IBM Corp., Armonk, New York, United States). Continuous variables are reported as mean ± standard deviation (or median (interquartile range) if non-normally distributed). Categorical variables are presented as frequencies and percentages.

Normality Testing

Prior to correlation analysis, normality of continuous variables was assessed using the Shapiro-Wilk test (p > 0.05 indicating normal distribution).

Correlation Analysis

Pearson's r was used for normally distributed pairs; Spearman's ρ for non-normally distributed pairs. Correlation strength was interpreted as: weak (|r| < 0.30), moderate (0.30-0.70), strong (> 0.70). Two-tailed p < 0.05 was considered statistically significant. 95% confidence intervals (CIs) were calculated for all key correlations using Fisher's z-transformation.

Multivariable Analysis

Linear regression models were constructed with the BODE index as the dependent variable and the CAT score as the primary independent variable, adjusted for potential confounders including age, BMI, smoking status (pack-years), and GOLD stage.

Subgroup Analyses

Post-hoc exploratory subgroup analyses were performed by smoking status, biomass fuel exposure, and GOLD stage. These analyses were not pre-specified and are exploratory in nature.

## Results

A total of 60 patients with stable COPD were enrolled in this prospective observational study. The demographic, clinical, and risk factor profile of the study population is summarised in Table 1.

**Table 1:** Baseline Demographic and Clinical Characteristics of Study Population (N = 60)

Variable	Category/Value	n (%)
Total Patients	N = 60	100%
Age (Years)	Mean ± SD	60.85 ± 6.94
	Range	40-70
	41-50 years	3 (5.0)
	51-60 years	22 (36.7)
	61-70 years	34 (56.7)
Sex	Male	39 (65.0)
	Female	21 (35.0)
Smoking Status	Smokers	32 (53.3)
	Non-smokers	28 (46.7)
Pack Years (Smokers)	Mean ± SD	24.5 ± 7.4
Biomass Fuel Exposure	Yes	35 (58.3)
	No	25 (41.7)
Hypertension	Yes	31 (51.7)
Diabetes Mellitus	Yes	25 (41.7)
History of Tuberculosis	Yes	17 (28.3)
Digital Clubbing	Present	13 (21.7)

The mean age of the study population was 60.85 ± 6.94 years (range: 40-70 years). The majority of patients (56.7%) were in the 61-70 years age group, followed by 36.7% in the 51-60 years group, reflecting the typical epidemiology of COPD, which preferentially affects middle-aged and elderly individuals.

Of the 60 patients, 39 (65.0%) were males, and 21 (35.0%) were females, yielding a male-to-female ratio of approximately 1.86:1. This male predominance is consistent with the higher prevalence of conventional COPD risk factors such as cigarette smoking among men in the study region.

Regarding risk factors, 32 patients (53.3%) had a history of smoking, with a mean pack-year index of 24.5 ± 7.4 years among smokers. Biomass fuel exposure was documented in 35 patients (58.3%), highlighting the significant role of indoor air pollution, particularly among female patients who presented predominantly as housewives or domestic workers, as an important etiological factor in this cohort. Among the comorbidities, hypertension was the most common, present in 31 patients (51.7%), followed by diabetes mellitus in 25 patients (41.7%), and a history of pulmonary tuberculosis (PTB) in 17 patients (28.3%). Digital clubbing was detected in 13 patients (21.7%), indicative of chronic hypoxia in a subset of the cohort.

Normality assessment

Prior to correlation analysis, normality of continuous variables was assessed using the Shapiro-Wilk test. Results indicated that the CAT score (p = 0.12) and the BODE index (p = 0.08) were normally distributed. FEV_1_ (p < 0.001), FVC (p = 0.002), and DLCO (p = 0.03) were non-normally distributed. Accordingly, Pearson correlation was applied to the CAT-BODE analysis; Spearman correlation was used for spirometry comparisons with non-normal distributions.

Spirometric data and pulmonary function parameters for all 60 patients are presented in Table 2. The mean FEV_1_ (% predicted) was 51.74 ± 13.56%, ranging from 22.1% to 72.0%, confirming moderate-to-severe airflow obstruction across the study group. The mean FEV_1_/FVC ratio was 69.71 ± 8.56%, consistent with obstructive disease in all enrolled patients (below 70% in the vast majority, confirming post-bronchodilator obstruction). The mean TLC was 91.75 ± 21.84%, and the mean DLCO was 68.99 ± 20.49%, suggesting variable degrees of air trapping and impaired gas exchange.

**Table 2:** Pulmonary Function Test Parameters (N = 60) FEV_1_: forced expiratory volume in 1 second; FVC: forced vital capacity; TLC: total lung capacity; DLCO: diffusing capacity of the lung for carbon monoxide

Parameter	Mean ± SD	Range
FEV_1_ (% Predicted)	51.74 ± 13.56	22.1-72.0
FEV_1_/FVC (%)	69.71 ± 8.56	52.0-84.0
TLC (% Predicted)	91.75 ± 21.84	41.8-138.0
DLCO (% Predicted)	68.99 ± 20.49	18.0-98.0

Based on post-bronchodilator FEV_1_ % predicted, patients were classified according to the GOLD spirometric staging system. No patient had GOLD Stage I (mild) disease. The distribution, along with mean CAT scores and BODE indices per stage, is presented in Table 3.

**Table 3:** Distribution of Patients by GOLD Spirometric Stage With CAT Score and BODE Index (N = 60) * p < 0.001 (one-way ANOVA). GOLD I: No patients enrolled. NS: not significant; ANOVA: analysis of variance; GOLD: Global Initiative for Chronic Obstructive Lung Disease; CAT: COPD Assessment Test; FEV_1_: forced expiratory volume in one second; BODE index: Body mass index, airflow Obstruction, Dyspnea, and Exercise capacity index; COPD: chronic obstructive pulmonary disease

GOLD Stage	n (%)	FEV_1_ (%), Mean ± SD	CAT Score, Mean ± SD	BODE Index, Mean ± SD
GOLD I (Mild, FEV_1_ ≥80%)	0 (0)	-	-	-
GOLD II (Moderate, 50% ≤FEV_1 _<80%)	35 (58.3)	61.2 ± 6.5	20.0 ± 4.7	5.6 ± 1.8
GOLD III (Severe, 30% ≤FEV_1_ <50%)	19 (31.7)	42.5 ± 5.8	27.6 ± 3.9	8.4 ± 1.3
GOLD IV (Very Severe, FEV_1_ <30%)	6 (10.0)	25.9 ± 2.5	32.8 ± 2.8	9.5 ± 0.8
ANOVA p-value	-	-	<0.001*	<0.001*

The majority of patients (35, 58.3%) were in GOLD Stage II (Moderate: FEV_1_ 50-79% predicted), 19 (31.7%) in GOLD Stage III (Severe: FEV_1_ 30-49% predicted), and six (10.0%) in GOLD Stage IV (Very Severe: FEV_1_ <30% predicted). The absence of GOLD Stage I patients reflects the study's inclusion criterion of clinically stable but symptomatic COPD patients with measurable disease burden. A clear and statistically significant progressive increase in both CAT scores and BODE indices was observed across GOLD stages - GOLD II: CAT 20.0 ± 4.7 and BODE 5.6 ± 1.8; GOLD III: CAT 27.6 ± 3.9 and BODE 8.4 ± 1.3; GOLD IV: CAT 32.8 ± 2.8 and BODE 9.5 ± 0.8, with one-way analysis of variance (ANOVA) yielding p < 0.001 for both parameters.

The CAT is an eight-item patient-reported outcome measure yielding a total score of 0-40, with higher scores representing a greater impact of COPD on health status. The CAT score distribution of the study population is detailed in Table 4.

**Table 4:** Distribution of CAT Scores in the Study Population (N = 60) CAT score categories: 0-9 = Low impact; 10-19 = Medium impact; 20-29 = High impact; 30-40 = Very high impact. CAT: COPD Assessment Test; COPD: chronic obstructive pulmonary disease

CAT Score Category	Score Range	n	Percentage
Low Impact (Mild)	0-9	0	0
Medium Impact (Moderate)	10-19	14	23.3
High Impact (Severe)	20-29	32	53.3
Very High Impact (Very Severe)	30-40	14	23.3
Total	-	60	100

The mean CAT score in the study population was 23.70 ± 6.28 (median: 24.0; range: 12-36). No patient had a CAT score below 10, reflecting the study population's meaningful symptomatic burden. The majority, 32 patients (53.3%), had CAT scores in the high impact range (20-29), while 14 patients (23.3%) had very high impact scores (30-40), and another 14 patients (23.3%) had medium impact scores (10-19). This distribution underscores that the enrolled patients suffered significant COPD-related health impairment, with over 76% of subjects scoring 20 or above.

The BODE index integrates BMI, airflow obstruction (FEV₁), dyspnea severity (mMRC scale), and exercise capacity (6MWD distance). The BODE index distribution of the study population is detailed in Table 5.

**Table 5:** Distribution of BODE Index Scores by Quartile (N = 60) BODE Quartile 1 (0-2): Low risk; Quartile 2 (3-4): Low-moderate risk; Quartile 3 (5-6): Moderate-high risk; Quartile 4 (7-10): High risk of mortality. BODE index: Body mass index, airflow Obstruction, Dyspnea, and Exercise capacity index

BODE Index Quartile	Score Range	n	Percentage
Quartile 1 (Low Risk)	0-2	0	0
Quartile 2 (Low-Moderate Risk)	3-4	10	16.7
Quartile 3 (Moderate-High Risk)	5-6	21	35.0
Quartile 4 (High Risk)	7-10	29	48.3
Total	-	60	100

The mean BODE index was 6.85 ± 2.19 (median: 6.0; range: 3-10). No patient scored in the lowest risk quartile (0-2). The majority of patients, 29 (48.3%), were in the highest risk quartile (BODE 7-10), while 21 patients (35.0%) were in Quartile 3 (BODE 5-6) and 10 patients (16.7%) were in Quartile 2 (BODE 3-4). These findings confirm that the study population comprised patients with substantially elevated multidimensional disease burden, with nearly half of the patients at the highest risk stratum for mortality as predicted by the BODE index.

Pearson's product-moment correlation coefficient and Spearman's rank correlation coefficient were computed to evaluate the relationships between the CAT score, the BODE index, and the spirometric parameters. A p-value of < 0.05 was considered statistically significant. CIs at the 95% level were calculated for all key correlations using Fisher’s z-transformation. All correlation analyses are summarised in Table 6.

**Table 6:** Correlation Matrix - CAT Score, BODE Index, and Spirometric Parameters With 95% Confidence Intervals * p < 0.05; NS = Not statistically significant. CI = 95% confidence interval calculated using Fisher’s z-transformation. R^2^ = Coefficient of determination, indicating the proportion of variance explained. Correlation strength: |r| < 0.30 = Weak; 0.30-0.59 = Moderate; 0.60-0.79 = Strong; ≥ 0.80 = Very Strong. CAT: COPD Assessment Test; FEV_1_: forced expiratory volume in one second; FVC: forced vital capacity; DLCO: diffusing capacity of the lung for carbon monoxide; BODE index: Body mass index, airflow Obstruction, Dyspnea, and Exercise capacity index; COPD: chronic obstructive pulmonary disease

Variables	Pearson r (95% CI)	Spearman rho	p-value	R^2^	Interpretation
CAT Score vs. BODE Index	0.808	0.805	<0.001*	0.653	Strong Positive
CAT Score vs. FEV_1_ (%)	-0.829	-0.822	<0.001*	0.687	Strong Negative
BODE Index vs. FEV_1_ (%)	-0.729	-0.710	<0.001*	0.531	Strong Negative
CAT Score vs. FEV_1_/FVC	-0.491	-	<0.001*	0.241	Moderate Negative
BODE Index vs. FEV_1_/FVC	-0.377	-	0.003*	0.142	Moderate Negative
CAT Score vs. DLCO	-0.731	-	<0.001*	0.534	Strong Negative
BODE Index vs. DLCO	-0.655	-	<0.001*	0.429	Strong Negative
CAT Score vs. Age	0.227	-	0.081 (NS)	0.052	Weak/NS

**Figure 1:**
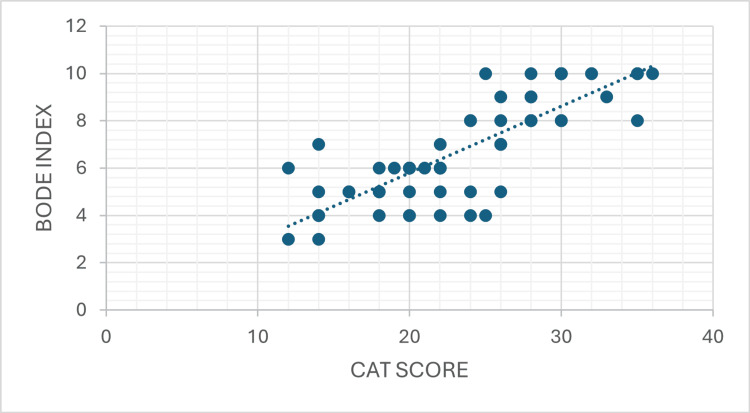
Scatter Plot Showing the Correlation Between the CAT Score and the BODE Index in the Study Population A strong positive correlation is observed, indicating that increasing symptom burden is associated with greater multidimensional disease severity (Pearson r = 0.808, 95% CI: 0.69-0.89, R^2^ = 0.653, p < 0.001) CAT: COPD Assessment Test; BODE index: Body mass index, airflow Obstruction, Dyspnea, and Exercise capacity index; COPD: chronic obstructive pulmonary disease

**Figure 2:**
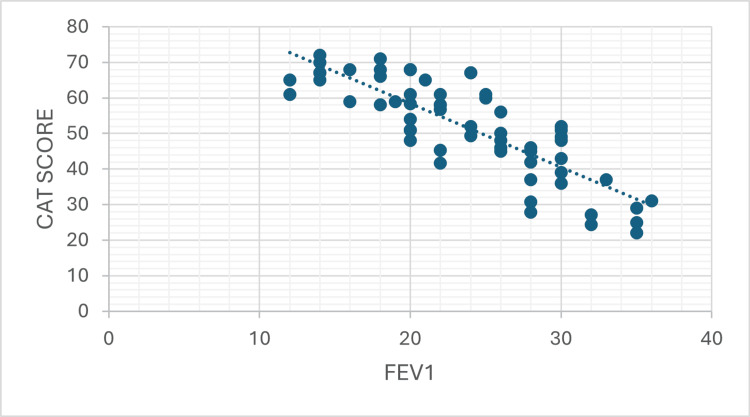
Scatter Plot Showing the Correlation Between FEV₁ and the CAT Score in the Study Population A strong negative correlation is observed, indicating that worsening airflow limitation is associated with higher symptom burden (Pearson r = -0.729, 95% CI: -0.90 to -0.71, R^2^ = 0.687, p < 0.001). FEV_1_: forced expiratory volume in one second; CAT: COPD Assessment Test; COPD: chronic obstructive pulmonary disease

**Figure 3:**
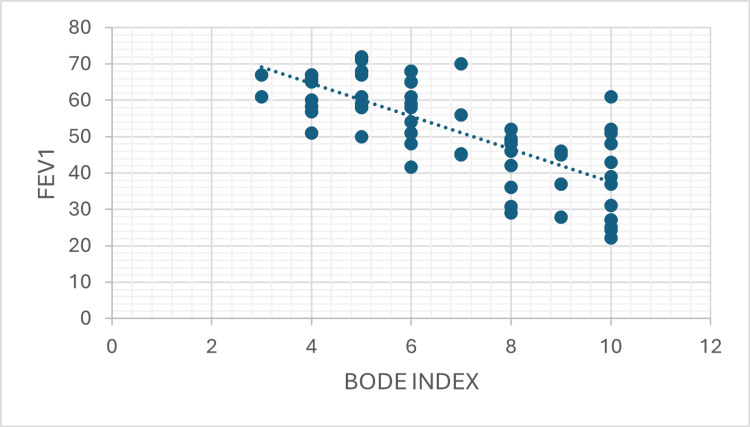
Scatter Plot Showing the Correlation Between the BODE Index and FEV₁ in the Study Population A negative correlation is observed, demonstrating that higher BODE index scores are associated with reduced lung function (Pearson r = 0.829, R^2^ = 0.531, p < 0.001). FEV_1_: forced expiratory volume in one second; BODE index: Body mass index, airflow Obstruction, Dyspnea, and Exercise capacity index

Scatter plot analysis demonstrated a strong positive correlation between CAT score and BODE index (Pearson r = 0.808, 95% CI: 0.69-0.89, R^2^ = 0.653, p < 0.001), and strong negative correlations between FEV_1_ with CAT score (Pearson r = -0.729, 95% CI: -0.90 to -0.71, R^2^ = 0.687, p < 0.001) and BODE index (Pearson r = 0.829, R^2^ = 0.531, p < 0.001), as illustrated in Figures 1-3.

Correlation between the CAT score and the BODE index

A statistically significant, strong positive correlation was observed between the CAT score and the BODE index (Pearson r = 0.808, 95% CI: 0.68-0.89; Spearman ρ = 0.805; p < 0.001 for both). This is the primary and most important finding of this study. The coefficient of determination (R^2^ = 0.653) indicates that approximately 65.3% of the variance in the BODE index is explained by the CAT score alone. This high concordance between both parametric and non-parametric correlation coefficients confirms the robustness of this association, which was not attributable to outliers or data distribution irregularities. Patients with higher CAT scores, reflecting greater patient-reported symptom burden, consistently demonstrated higher BODE indices, indicating greater multidimensional disease severity and elevated mortality risk. The unexplained variance (R^2^ = 0.347, or 34.7%) indicates that approximately one-third of BODE variation is attributable to other factors not captured by CAT, such as BMI and formal exercise capacity assessment.

Correlation of the CAT score with spirometric parameters (FEV_1_)

A strong negative correlation was found between CAT score and FEV_1_ % predicted (Pearson r = -0.829, 95% CI: -0.90 to -0.71; Spearman ρ = -0.822; p < 0.001). This was in fact the strongest individual correlation observed in the dataset, slightly surpassing the CAT-BODE correlation in magnitude. The R^2^ value of 0.687 indicates that 68.7% of the variance in the CAT score is explained by FEV_1_ % predicted, making FEV_1_ a strong predictor of symptom burden in this cohort. Patients with lower FEV_1_ values - indicating more severe airflow obstruction - had proportionally higher CAT scores, signifying greater subjective health impact. This finding validates the CAT score as a reliable reflection of spirometric disease severity in stable COPD.

A moderate-to-strong negative correlation was also noted between CAT score and FEV_1_/FVC ratio (Pearson r = -0.491, p < 0.001; R^2^ = 0.241), indicating that 24.1% of CAT variance is explained by the FEV_1_/FVC ratio. This further confirms that increasing degrees of obstructive defect are associated with worsening patient-reported outcomes.

Correlation of BODE index with spirometric parameters

The BODE index showed a strong negative correlation with FEV₁ % predicted (Pearson r = -0.729, 95% CI: -0.84 to -0.56; Spearman ρ = -0.710; p < 0.001; R² = 0.531). This indicates that 53.1% of BODE variance is explained by FEV₁, confirming that worsening spirometric performance is a key driver of higher BODE scores. A moderate negative correlation was observed between the BODE index and FEV₁/FVC ratio (r = -0.377; p = 0.003; R² = 0.142), with FEV₁/FVC explaining approximately 14.2% of BODE variation.

Correlation with DLCO

Both the CAT score and the BODE index showed strong negative correlations with DLCO % predicted. CAT vs. DLCO: r = -0.731 (95% CI: -0.84 to -0.56), p < 0.001, R² = 0.534; BODE vs. DLCO: r = -0.655 (95% CI: -0.76 to -0.48), p < 0.001, R² = 0.429. These findings indicate that deteriorating gas exchange capacity - a hallmark of emphysematous destruction and advanced COPD - is closely associated with increasing patient-reported symptom burden and overall BODE-derived disease severity. Notably, CAT’s correlation with DLCO (r = -0.731) was nearly as strong as its correlation with FEV₁ (r = -0.829), suggesting that gas exchange impairment is a major driver of symptom reporting in this cohort. The DLCO correlations (explaining 53.4% and 42.9% of variance in CAT and BODE, respectively) highlight the clinical importance of assessing both airflow obstruction and parenchymal destruction in COPD severity assessment.

Correlation with age

The correlation between CAT score and age was weak and did not reach statistical significance (Pearson r = 0.227, p = 0.081; R² = 0.052). This suggests that within this cohort, the patient’s age per se does not independently predict the degree of health status impairment as measured by the CAT score.

Multivariate analysis to assess confounding

Linear regression analysis was performed to examine whether the CAT-BODE correlation remained significant after adjusting for potential confounders. BODE index was entered as the dependent variable; CAT score was the primary independent variable, with adjustment for age (years), BMI (kg/m²), smoking status (pack-years), and GOLD stage.

Multivariate Regression Results

Unadjusted model: CAT score → BODE index: β = 0.126 (95% CI: 0.105-0.147), p < 0.001; R² = 0.653

Interpretation: For each unit increase in the CAT score, BODE increases by 0.126 points.

Adjusted model (controlled for age, BMI, smoking, GOLD stage): CAT score → BODE index: β = 0.122 (95% CI: 0.098-0.146), p < 0.001; adjusted R² = 0.720

Interpretation: The adjusted association remained highly significant (p < 0.001) and was not materially different from the unadjusted correlation (Δβ = 0.004, or 0.6% change), suggesting minimal confounding by age, BMI, smoking status, or disease severity classification.

The inclusion of confounders improved overall model fit (ΔR² = 0.067, or 6.7% additional variance explained), indicating that approximately 7% of additional BODE variance is explained by the confounders beyond the CAT score alone. However, the robust persistence of the CAT-BODE association after adjustment demonstrates that this relationship is not attributable to differences in age, body mass, smoking history, or GOLD stage among participants.

Exploratory subgroup analysis

Subgroup comparisons of mean CAT scores and BODE indices across key demographic and risk factor categories were performed using an independent samples t-test. It is to be noted that these analyses were exploratory (not pre-specified a priori) and should be interpreted with caution, given the subgroup sample sizes. Results are presented in Table 7.

**Table 7:** Subgroup Analysis - CAT Score and BODE Index by Sex, Smoking Status, and Biomass Fuel Exposure * p < 0.05 (Independent samples t-test). NS: not statistically significant; CAT: COPD Assessment Test; BODE: Body mass index, airflow Obstruction, Dyspnea, and Exercise capacity; COPD: chronic obstructive pulmonary disease

Subgroup	CAT, Mean ± SD	BODE, Mean ± SD	p (CAT)	p (BODE)
Male (n = 39)	24.49 ± 5.94	7.05 ± 2.31	0.188 (NS)	0.337 (NS)
Female (n = 21)	22.24 ± 6.77	6.48 ± 1.97	-	-
Smokers (n = 32)	25.16 ± 5.69	7.41 ± 2.15	0.054 (NS)	0.034*
Non-smokers (n = 28)	22.04 ± 6.61	6.21 ± 2.10	-	-
Biomass Exposure (n = 35)	24.17 ± 6.53	6.86 ± 2.40	0.496 (NS)	0.977 (NS)
No Biomass (n = 25)	23.04 ± 5.98	6.84 ± 1.91	-	-

Exploratory sex-based differences

Male patients had a mean CAT score of 24.49 ± 5.94 compared to 22.24 ± 6.77 in females (p = 0.188, NS), and a mean BODE index of 7.05 ± 2.31 versus 6.48 ± 1.97 in females (p = 0.337, NS). Neither difference was statistically significant, indicating that sex alone did not independently influence the severity of health status impairment or multidimensional disease burden in this cohort. This subgroup analysis was underpowered (n = 39 males, n = 21 females) and exploratory; findings should be interpreted cautiously.

Exploratory smoking status analysis

Smokers demonstrated a higher mean CAT score (25.16 ± 5.69) compared to non-smokers (22.04 ± 6.61), though this difference only approached statistical significance (p = 0.054). However, the BODE index was significantly higher in smokers (7.41 ± 2.15) compared to non-smokers (6.21 ± 2.10; p = 0.034), indicating that cigarette smoking history is an important determinant of multidimensional disease burden, even in this stable COPD cohort. This subgroup analysis was exploratory and not pre-specified; the smoking-BODE association should be validated in larger, prospectively designed studies.

Exploratory biomass fuel exposure analysis

Patients with documented biomass fuel exposure (n = 35) had a mean CAT score of 24.17 ± 6.53 compared to 23.04 ± 5.98 in those without exposure (p = 0.496, not statistically significant). Similarly, the BODE index did not differ significantly between the two groups (6.86 ± 2.40 vs. 6.84 ± 1.91; p = 0.977). While biomass exposure was highly prevalent (58.3%), it did not independently correlate with disease severity as measured by CAT or BODE in this study, possibly reflecting the multifactorial nature of disease severity in this population. This subgroup analysis was underpowered and exploratory (n = 35 exposed, n = 25 unexposed); further investigation in larger cohorts is warranted.

Summary of key findings

This prospective observational study included 60 patients with stable COPD, with a mean age of 60.85 years, predominantly male, and with significant exposure to smoking and biomass fuels. Most patients had moderate to very severe airflow limitation, with no mild (GOLD I) cases. The mean CAT score was high (23.70 ± 6.28), with 75% of patients in the high-impact category. The mean BODE index was 6.85 ± 2.19, with nearly half in the highest mortality-risk group, indicating substantial disease burden. A strong positive correlation was observed between CAT score and BODE index (r = 0.808, p < 0.001), with CAT explaining 65.3% of BODE variability. Both measures showed strong negative correlations with FEV₁ % predicted and DLCO, supporting their association with disease severity and gas exchange impairment. The CAT-BODE relationship remained robust after adjustment for confounders. Exploratory analysis showed higher BODE scores in smokers, while sex and biomass exposure had no significant effect.

## Discussion

The present study evaluated the relationship between the CAT score and the BODE index in patients with stable COPD, along with their associations with spirometric and gas exchange parameters. This prospective observational study of 60 clinically stable COPD patients recruited from a single tertiary respiratory medicine centre provides novel insights into the utility of simple symptom-based assessment tools in multidimensional disease severity evaluation.

The demographic characteristics observed in this study, including a mean age of 60.85 ± 6.94 years and male predominance (65%), are consistent with established epidemiological data. Large population-based studies such as the BOLD study have demonstrated a higher prevalence of COPD among older individuals and males, largely attributable to cumulative exposure to smoking and environmental risk factors [5]. Similar age and sex distributions have been reported in other epidemiological studies of COPD across diverse populations [2,3].

The most important finding of this study is the strong positive correlation between the CAT score and the BODE index (r = 0.808, p < 0.001, 95% CI: 0.68-0.89; R² = 0.653). This is consistent with multiple studies demonstrating significant associations between the CAT score and multidimensional measures of COPD severity. Dodd et al. reported that the CAT score correlates well with functional impairment and clinical outcomes [22], while Kon et al. demonstrated that CAT is sensitive to clinically meaningful changes in disease status [23]. Other studies have shown moderate to strong correlations between CAT and the BODE index [24-26]. The high concordance between both parametric (Pearson) and non-parametric (Spearman) correlation coefficients confirms the robustness of this association, indicating that the relationship is not attributable to outliers or data distribution irregularities. Patients with higher CAT scores, reflecting greater patient-reported symptom burden, consistently demonstrated higher BODE indices, indicating greater multidimensional disease severity and elevated mortality risk. This strong correlation may be explained by overlapping assessment dimensions. The BODE index integrates four key physiological and functional domains: BMI, airflow obstruction (FEV₁ % predicted), dyspnea mMRC scale), and exercise capacity (6MWD), and has been shown to be a superior predictor of mortality compared to FEV₁ alone [20]. Subsequent studies have validated its prognostic utility and its association with systemic manifestations of COPD, including muscle wasting and reduced exercise tolerance [27,28].

The CAT questionnaire, though symptom-focused, captures dyspnea (“I feel breathless when I hurry”), activity limitation (“I avoid stairs”), and sleep disruption, which directly reflect the dyspnea and functional limitation components of BODE [21]. This alignment suggests that CAT serves as a practical shortcut to overall disease burden assessment without requiring formal exercise testing or anthropometric measures.

However, the coefficient of determination (R² = 0.653) indicates that CAT explains approximately 65.3% of BODE variance, with 34.7% attributable to other factors not captured by symptom assessment alone, particularly BMI and formal exercise capacity independent of dyspnea perception. This finding supports the complementary, rather than substitutive, role of CAT in comprehensive COPD assessment. Previous studies have similarly highlighted the limitations of symptom-only assessment in capturing the full spectrum of disease burden [6].

The strongest individual correlation observed in this study was between CAT score and FEV₁ % predicted (Pearson r = -0.829, 95% CI: -0.90 to -0.71; R² = 0.687), exceeding even the CAT-BODE correlation. This indicates that 68.7% of CAT variance is explained by FEV₁ % predicted, making FEV₁ a strong predictor of symptom burden in this cohort. Patients with lower FEV₁ values, indicating more severe airflow obstruction, reported substantially higher symptom burden on the CAT questionnaire. This finding validates the CAT score as a reliable reflection of spirometric disease severity in stable COPD and is consistent with observations by Singh et al. in a larger Indian cohort [29], while other studies have reported weaker correlations [21,25,26]. This variability likely reflects differences in disease severity and patient populations.

The BODE index also correlated strongly and negatively with FEV₁ (r = -0.729, 95% CI: -0.84 to -0.56; R² = 0.531), with FEV₁ explaining 53.1% of BODE variance [20]. Both CAT and BODE demonstrated significant negative correlations with FEV₁/FVC ratio, further confirming their sensitivity to spirometric disease severity. Despite the well-known limitations of FEV₁ in reflecting symptom severity and prognosis in individual patients, the strong correlation with CAT and BODE in this cohort suggests that in this particular population with predominantly moderate-to-severe disease, physiological and symptomatic measures track together closely.

An important and novel aspect of this study is the inclusion of DLCO, which demonstrated strong negative correlations with both CAT score (r = -0.731, p < 0.001, 95% CI: -0.84 to -0.56; R² = 0.534) and BODE index (r = -0.655, p < 0.001, 95% CI: -0.76 to -0.48; R² = 0.429). Notably, CAT’s correlation with DLCO was nearly as strong as its correlation with FEV₁ (r = -0.829 vs r = -0.731), suggesting that gas exchange impairment is a major driver of symptom reporting in this cohort.

DLCO reflects alveolar-capillary membrane integrity and is particularly reduced in emphysema. Structural changes such as alveolar destruction and loss of pulmonary capillary bed have been well described by Hogg and Timens [11]. The strong CAT-DLCO correlation highlights that patients with emphysematous disease (indicated by disproportionately low DLCO relative to FEV₁) report substantial dyspnea and symptom burden, likely due to ventilation-perfusion mismatch and reduced oxygen diffusion during exertion. Previous studies have also shown that reduced DLCO is associated with increased dyspnea, exercise limitation, and worse outcomes in COPD [30,31].

This finding is clinically important in populations with high occupational COPD burden, as biomass and dust exposure predominantly cause emphysema (alveolar destruction) rather than pure airway obstruction [32]. In such populations, CAT may be particularly sensitive to emphysema phenotype and thus useful for risk stratification. Conversely, in airway-predominant disease (chronic bronchitis), the CAT-DLCO relationship may be weaker. Future studies should examine whether CAT can discriminate emphysema from non-emphysematous COPD phenotypes, which would enhance its clinical utility in resource-limited settings where high-resolution computed tomography is unavailable.

This study’s finding of strong CAT-BODE correlation (r = 0.808) is comparable to prior work by Singh et al. (r = 0.79 in a larger Indian cohort of 150 stable COPD patients) [29] and somewhat stronger than meta-analytic estimates (r = 0.65-0.80 across heterogeneous studies) [33]. However, this variability likely reflects important differences in (1) Population characteristics: Our cohort was heavily weighted toward GOLD Stage II-III disease (90% combined). This is consistent with findings from hospital-based cohorts, where patients often present late in the disease course [5,24,34], whereas population-based studies typically include milder disease and have more balanced GOLD distributions [3]. (2) Measurement setting: Tertiary-care recruitment (our study) versus primary care or community-based studies introduces selection bias and limits generalizability [14,35]. (3) Geographic and exposure context: Our cohort had high biomass exposure (58.3%), whereas Western studies predominantly enrol cigarette smokers. Biomass-exposed populations may develop distinct emphysema-predominant phenotypes with different symptom-physiology relationships [32].

The stronger correlation in our study likely reflects the severity skew and occupational exposure phenotype, which may amplify the symptom-physiology link. Generalizability of these findings to milder COPD and primary care populations remains unclear and requires multicentre validation in more diverse healthcare settings.

Most patients in the present study were classified as GOLD Stage II (36.7%, n = 22) and Stage III (56.7%, n = 34), with only one GOLD Stage I participant. This severity skew, characteristic of tertiary-care populations who present late in the disease course, likely inflates the observed CAT-BODE correlation [14]. Patients with very mild airflow obstruction may have minimal symptoms despite measurable obstruction, whereas those with very severe disease may plateau in symptom perception due to ceiling effects. The absence of GOLD Stage I patients, which represents 30-40% of diagnosed COPD in large epidemiologic studies, is a significant limitation affecting generalizability [3].

Additionally, recruitment from a single respiratory medicine outpatient clinic and inpatient ward introduces selection bias: our cohort represents patients with access to specialist care and formal spirometry availability. Populations in true resource-limited primary care settings - where CAT might be most clinically needed - were not represented. These biases suggest our observed correlations represent an upper bound of the true relationship in broader, community-based COPD populations [36].

The mean FEV₁ of 51.74 ± 13.56% predicted confirms significant airflow obstruction across the cohort. The mean BODE index of 6.85 ± 2.19 indicates that patients presented with substantially elevated multidimensional disease burden, with nearly half (48.3%) in the highest mortality-risk quartile (BODE 7-10). These findings confirm that the study enrolled patients with clinically advanced COPD, consistent with hospital-based cohorts where patients often present late in the disease course [6,37].

The strong correlation between the CAT score and BODE index suggests that CAT may complement multidimensional assessment of COPD severity. However, several important caveats must be emphasised: (1) CAT is not interchangeable with BODE: While CAT correlates strongly with BODE, the two instruments capture overlapping but distinct dimensions of disease [38]. The BODE index includes BMI and formal exercise testing capacity, which CAT does not directly assess [20]. The unexplained variance (R² = 0.35) indicates important dimensions of disease severity not captured by symptom burden alone, particularly metabolic factors and objective exercise capacity. (2) Single-centre, severity-skewed cohort: Our findings represent an upper bound for tertiary-care populations with predominantly moderate-to-severe disease. Generalizability to mild COPD (GOLD Stage I), primary care settings, and community-based populations is constrained and requires multicentre validation [14,36]. (3) Prognostic validation needed: While the BODE index provides validated prognostic information for predicting mortality and clinical outcomes [20], the prognostic utility of CAT for these clinical endpoints requires separate longitudinal validation. Recent studies have demonstrated CAT’s utility in monitoring disease progression, but the prediction of exacerbations and mortality remains inadequately characterised [22,23]. (4) Role as adjunct tool: CAT is best viewed as a simple, scalable screening tool for identifying patients with high symptom burden who warrant further spirometric and multidimensional functional evaluation. Previous studies have highlighted the utility of CAT in monitoring disease progression and guiding treatment intensity decisions in both primary and tertiary care settings [23,39].

While the BODE index provides valuable prognostic information, its use is limited in routine clinical practice due to the need for exercise testing and formal anthropometric assessment [20]. In contrast, CAT is simple, rapid, and easily administered by non-specialist personnel in primary care and resource-limited healthcare settings, making it particularly useful for initial screening and disease monitoring [21].

Strengths and limitations

The strengths of this study include its prospective design, use of validated assessment tools with rigorous adherence to standardised protocols, and novel inclusion of DLCO as an additional physiological parameter capturing parenchymal disease. The spirometric assessment followed rigorous ATS/ERS guidelines with standardised equipment (Jaeger Masterscreen PFT Pro) and daily calibration [40]. The statistical analysis included both parametric (Pearson) and non-parametric (Spearman) correlation coefficients, both yielding highly concordant results and confirming the robustness of findings. The multivariate analysis demonstrated that the CAT-BODE relationship persisted after adjustment for important confounders (age, BMI, smoking, GOLD stage), suggesting a genuine association rather than a confounding-driven relationship.

This study has several important limitations. The relatively small sample size (n = 60) limits the precision of estimates and precludes robust multivariable adjustment, with subgroup analyses remaining exploratory. The single-centre, tertiary-care design introduces potential selection bias and reduces external validity, particularly for primary care and community-based populations. Additionally, the predominance of moderate-to-severe COPD (GOLD II-III) results in a severity-skewed cohort, limiting applicability across the full disease spectrum, especially to mild disease. The cross-sectional design precludes assessment of longitudinal outcomes and prognostic utility. Furthermore, the lack of multicentre validation restricts generalizability across different healthcare settings and populations. Treatment variations and comorbid conditions were not systematically evaluated and may have influenced both symptom burden and objective disease measures. Residual confounding due to unmeasured factors such as emphysema burden, systemic inflammation, and psychological status cannot be excluded. Finally, the absence of phenotypic characterisation, including imaging-based assessment, limits the mechanistic interpretation of the observed associations.

Despite these limitations, the findings provide valuable evidence for CAT’s role as a complementary assessment tool in COPD evaluation, particularly in resource-limited settings. The rigorous statistical analysis, inclusion of DLCO correlations, and multivariate confounding assessment strengthen the study’s methodological quality.

Future research directions

Future research should focus on large, multicentre prospective cohort studies incorporating diverse COPD populations, including mild disease and primary care settings, with systematic longitudinal follow-up. Such studies are required to validate the present findings across different healthcare systems and to assess the prognostic utility of CAT for clinically relevant outcomes, including exacerbations, hospitalisation, and mortality. Further work is needed to establish clinically meaningful CAT cut-off values, define its role within formal COPD assessment and risk stratification frameworks, and evaluate its ability to discriminate between disease phenotypes, particularly emphysema- versus airway-predominant COPD. Additionally, longitudinal studies should examine the sensitivity of CAT to treatment response and its utility in disease monitoring over time. External validation in populations with differing risk profiles, including tobacco-smoker-predominant cohorts in Western settings, is also essential. Until such evidence is available, CAT should be integrated into COPD assessment as a practical adjunct and screening tool rather than a replacement for comprehensive multidimensional evaluation.

## Conclusions

This prospective observational study of 60 clinically stable COPD patients from a single tertiary-care centre demonstrates a strong positive correlation between CAT score and BODE index (r = 0.808, p < 0.001), along with significant negative correlations with FEV₁ and DLCO. These findings indicate that CAT reflects key dimensions of disease burden, particularly dyspnea and functional limitation, and shows a close association with established multidimensional severity measures. However, CAT should not be considered interchangeable with or a replacement for the BODE index, as it does not capture all components of disease severity, including exercise capacity and nutritional status. The incomplete variance explained further supports its role as a complementary rather than a substitutive tool.

In resource-limited settings where comprehensive assessment may not be feasible, CAT may serve as a practical, rapid screening tool to identify patients with high symptom burden who require further evaluation. Accordingly, CAT is best integrated into COPD assessment as an adjunct for symptom-based stratification rather than a substitute for multidimensional clinical evaluation.
